# Supplementary material for: Are carbon emissions trading and green financial instruments synergistic? -Comprehensive quantitative research based on content analysis
Source: PLoS One. 2024 Mar 7;19(3):e0298601. doi: 10.1371/journal.pone.0298601 (PMC10919601; doi:10.1371/journal.pone.0298601)
Supplement: S1 Data — (ZIP) [file pone.0298601.s001.zip › Data/地级市Z_F工作报告.zip!/─┌├╔╣┼/░═╤σ─╫╢√╩╨/└┤╫╘╣1⁄2╓┌║┼í╛┬φ┐╦╩2╛▌═°í┐_2013.docx]

各位代表：

　　现在，我代表市人民政府向大会报告工作，请予审议，并请市政协委员和列席会议的同志们提出意见。

　　一、过去五年的工作

　　过去五年，在市委的坚强领导下，我们团结依靠全市各族人民，紧紧抓住国家实施西部大开发、促进内蒙古经济社会又好又快发展等历史机遇，有效应对国际金融危机等一系列风险挑战，圆满完成了市二届人大一次会议确定的目标任务，谱写了巴彦淖尔发展史上亮丽的篇章。

　　——综合经济实力大幅提升。预计地区生产总值由2007年的341.6亿元增加到2012年的812亿元，年均增长14.8%。地方财政总收入由45.5亿元增加到90.4亿元，年均增长14.7%。累计完成固定资产投资2798亿元，年均增长20.9%。社会消费品零售总额由72.7亿元增加到169亿元，年均增长18.4%。城镇居民人均可支配收入由10360元增加到18823元，年均增长12.7%。农牧民人均纯收入由5435元增加到10716元，年均增长14.5%。

　　——产业转型实现重大突破。预计三次产业结构由22∶49∶29演进为18∶59∶23，工业比重由40.8%提高到50.4%，实现了农牧业主导型经济向工业引领型经济的重大转变。优势特色产业进一步巩固，新型煤化工、氯碱化工破题起步，新能源、装备制造等新兴产业发展迅速。培育和引进了河套酒业、巴运、紫金、神华、联邦、包钢等一批大企业，4个工业园区跨入百亿园区行列。粮食总产量达到58.6亿斤，牲畜饲养量突破1700万头（只），巴美肉羊等农畜产品通过国家地理标志保护登记。第三产业增加值达到187亿元，年均增长12.2%。

　　——城乡面貌发生显著变化。确立了“一城三区一组团”的城市发展新格局，城镇化率由43.6%提高到51%。临河新区基本成型，旧城区面貌大为改观，双河区启动建设。县城和中心集镇建设卓有成效，完成苏木乡镇基础设施建设项目25个，新农村新牧区建设走在全区前列。交通、水利、电力等基础设施更加完备，巴彦淖尔机场通航运营，临策、甘泉铁路竣工通车，在全区率先实现苏木乡镇全部通油路。完成林业生态建设416万亩，河套灌区森林覆盖率由10.1%提高到15.7%。节能减排任务如期完成。

　　——民生和社会事业全面发展。各级财政用于民生的投入达453.7亿元，占财政总支出的比重由62.6%提高到71.5%。城乡就业进一步扩大，社会保障标准大幅提高，基本养老、医疗保险实现制度全覆盖，城乡低保应保尽保。实施棚户区改造321万平方米，建设保障性住房3.76万套，完成农村牧区危房改造3.3万户。城乡免费义务教育全面实现，105万平方米校安工程竣工投用，市中学新校区建成使用。公共医疗卫生服务体系基本建立。创作推出了《河套长烟》、《阴山·古歌》等一批优秀文化艺术作品，5个旗县博物馆建成开馆，河套文化的影响力和知名度日益扩大。科技、扶贫和人口计生等事业全面进步。

　　——改革开放迈出坚实步伐。新一轮政府机构改革全面完成，事业单位改革稳步实施。行政审批、金融财税、医药卫生和文化体制改革不断深化。向北开放取得历史性突破，甘其毛都口岸实现双边常年开放，过货量突破1200万吨，是2007年的6倍，成为自治区过货量最大的公路口岸。招商引资和区域合作广泛开展，引进国内到位资金1047亿元，实际利用外资2.6亿美元。

　　——民主法制和政府自身建设不断加强。自觉接受人大依法监督和政协民主监督，共办理人大代表建议681件，政协委员提案1390件，办结率均达到100%，满意率达98%以上。投资1.6亿元的市政务中心建成投用，持续开展效能监察和行风评议，政务服务水平不断提高。精神文明创建活动广泛开展，连续举办四次全市两个文明建设现场经验交流会。社会管理创新扎实推进，民族团结、社会稳定、边疆安宁的局面进一步巩固。

　　各位代表，刚刚过去的2012年，是我们经受重大考验的一年。面对复杂多变的经济形势和严重自然灾害，全市上下以“总干”精神为引领，以迎接全区第九次精神文明建设经验交流会为契机，坚持“一产做精、二产做强、三产做活、城市做美、生态做优、民生做实”，全力以赴保增长、惠民生、促和谐，各项工作取得了新的成绩。

　　一年来，我们加快发展现代农牧业，新增设施农业5.16万亩，建设规模化养殖场310个，农牧业抗灾能力明显增强，生产效益显著提升，农牧民人均纯收入增长13%。一年来，我们千方百计保工业、抓项目，促进工业经济企稳回升。预计规模以上工业增加值增长15%。神华煤焦化、中电投光伏发电等项目建成试运行，圣泉生物质能源、河套酒业技改扩建、黄河工贸PVC等项目开工建设。巴彦淖尔经济技术开发区升级为国家级开发区。一年来，我们着力抓好第三产业发展，纳林湖、奈伦湖等旅游景区建设初具规模，秋林物流园区开工建设，红美建材家居生活广场建成运营。金融机构存贷款余额均突破580亿元，创历史新高。一年来，我们围绕做好“水、绿、文化”三篇文章，继续掀起城镇大建设、大改造热潮。黄河、总干渠和各旗县区、农垦局湿地恢复一期工程基本建设完成，文博中心建成投用，新华街综合改造、主要街路绿化亮化美化等工程全面实施，城市形象明显改观。一年来，我们切实加强生态和基础设施建设，投资16.4亿元完成林业生态建设81.6万亩，是近年来投入最多、力度最大、效果最好的一年。临河黄河大桥竣工通车，279公里黄河堤防公路全线贯通，总投资15亿元的河套灌区排水改造工程启动实施。一年来，我们下大力气抓好民生和社会建设，“十件民生实事”全部完成，“十项民生工程”加快推进。机关工作人员津补贴月人均增加500元，事业单位绩效工资改革实施到位。河套大学升本成功。报业传媒改革顺利完成。阴山岩刻列入中国世界文化遗产预备名单。电视剧《我叫王土地》荣获中国电视金鹰奖。9个高标准社区服务中心基本建成。认真对待和解决群众来信来访，扎实开展社会矛盾排查化解，社会保持和谐稳定。特别是面对历史罕见的洪涝灾害，全市上下万众一心，众志成城，取得了抢险救灾和灾后重建的全面胜利。回顾过去一年取得的成绩，我们深感来之不易，又为之欣慰和激动，在困难和挑战面前，广大干部群众经受了锻炼和考验，“总干”精神在全市人民身上再次被激发、再次得到展现和升华！各位代表，五年的变化有目共睹，五年的成就令人振奋。这是自治区党委、政府和市委正确领导的结果，是市人大、政协有效监督和大力支持的结果，也是各族各界干部群众同心同德、共同奋斗的结果。在此，我代表市政府，向全市人民，向所有为巴彦淖尔建设和发展作出贡献的同志们、朋友们，表示衷心的感谢和崇高的敬意！

　　在看到成绩的同时，我们也清醒认识到，经济社会发展中还存在不少困难和问题。主要是：经济总量小、结构不尽合理，产业层次依然不高，财政持续增收难度加大；中心城市建设相对滞后，生态环境脆弱的状况还没有得到根本改变；征地拆迁、涉法涉诉等领域的社会矛盾增多，社会管理有待进一步加强；城乡居民收入偏低，部分群众生活还比较困难；政府职能转变还不到位，公共服务、公共设施与人民群众的需求还有不小差距，等等。在继往开来的新征程上，我们有信心、有决心解决这些问题，绝不辜负全市人民的期望。

　　二、今后五年的工作

　　今后五年，是推进富民强市、赶超崛起的关键时期，也是大有作为的重要战略机遇期。党的十八大确定了全面建成小康社会和深化改革开放的总目标，明确了中国特色社会主义“五位一体”的总布局，描绘了社会主义现代化建设的宏伟蓝图，为我们赶超崛起指明了前进方向。国家实施新一轮西部大开发战略，促进内蒙古经济社会又好又快发展，为我们赶超崛起提供了有力的政策支持。我市多年形成的发展能量蓄势待发，全市人民在“总干”精神引领下，凝心聚力谋发展的精气神空前高涨，为我们赶超崛起增添了前所未有的力量源泉。只要我们牢牢把握机遇，沉着应对挑战，完全可以在新一轮发展中赢得主动、赢得优势、赢得未来。

　　今后五年政府工作的总体思路是：深入学习贯彻党的十八大精神，以邓小平理论、“三个代表”重要思想、科学发展观为指导，融注“总干”之魂，紧扣“崛起”主题，按照“五位一体”总布局，加快工业化、信息化、城镇化和农牧业现代化进程，推进生态文明和基础设施建设，切实保障和改善民生，促进文化繁荣发展，深化改革开放和科技创新，全力实施“五大工程”，建设富裕、美丽、幸福、文明、活力巴彦淖尔。

　　（一）实施产业强市工程，建设富裕巴彦淖尔。坚持发展第一要务不动摇，继续做大经济总量，调整优化经济结构，一产提效，二产提质，三产提档，大力发展县域经济，不断增强综合实力。到2017年，地区生产总值突破1200亿元，地方财政总收入突破130亿元，均比2010年翻一番，经济发展总体达到自治区中等以上水平。加快新型工业化进程。坚持提升传统产业与培育新兴产业并重，壮大骨干企业与发展中小企业并重，以项目为抓手，以园区为载体，以构建产业集群为重点，推动工业扩量提质增效。到2017年，形成2000万吨以上煤焦化、300万吨以上PVC生产能力，有色和黑色金属冶炼加工能力分别达到100万吨、500万吨以上，风电装机容量达到1000万千瓦、光伏发电100万千瓦，以生物制药、装备制造为代表的新兴产业成为重要经济增长点。大力发展现代农牧业。加快调整农牧业结构，积极发展高效种养业，建设特色农畜产品加工出口基地，培育壮大巴美肉羊、乌拉特黑山羊、河套面粉、二狼山白山羊绒、康尔徕、圣牧高科等一批农畜产品知名品牌，提升农牧业经济效益，推动农牧业大市向农牧业强市转变。到2017年，设施农业达到40万亩以上，规模化养殖比重提高到85%以上。拓展提升第三产业。加大旅游资源的整合和开发力度，推动旅游与文化融合发展，打造黄河旅游、草原旅游、田园旅游品牌，加快重点旅游景区建设，使我市成为自治区西部独具特色的旅游目的地。培育壮大物流、金融、商贸、信息等服务业，提高服务业水平。

　　（二）实施环境优化工程，建设美丽巴彦淖尔。美丽发展是人民群众的共同期待。把生态文明建设融入经济、政治、文化、社会建设全过程，大力改善城乡环境，建设天蓝、地绿、水净的美好家园。按照“一城三区一组团”城市发展规划，加快建设百万人口区域中心城市。加速推进双河区建设，3至5年内17.4平方公里的核心区框架基本成型。加强临河主城区、朔方区建设，推进陕坝镇与主城区组团发展。全面提升县城和集镇建设水平。实施湿地保护和园林绿化工程，新建一批彰显河套文化内涵的标志性建筑，打造草原水城、西北绿城、河套文化名城。到2017年，全市城镇化率达到60%，基本实现农牧业社会形态向工业社会形态的转变。加大城乡统筹力度，推进新农村新牧区建设，实施村庄综合改造工程，两年内完成全部嘎查村规划编制，建成一批设施完善、整洁优美的农牧民新村。强化交通、水利、电力、通讯等基础设施建设，提高支撑保障能力。以构筑祖国北疆重要生态屏障为目标，推进乌梁素海、乌兰布和沙漠、乌拉特草原生态综合治理，抓好河套平原绿化，展现秀美田园风光，全市植被覆盖率达到50%，河套灌区森林覆盖率达到20%。加强资源节约和环境保护，推进绿色发展、循环发展、低碳发展，保护我们的环境，让青山绿水长在、美丽与发展共赢。

　　（三）实施民生改善工程，建设幸福巴彦淖尔。人民对美好生活的向往，就是我们的奋斗目标。要始终把改善民生放在一切工作的首位，解决好群众最关心、最直接、最现实的利益问题，让全市人民生活得更加幸福。落实各项增收政策，切实增加城乡居民收入，努力实现“两个同步 、两个提高”。到2017年，城镇居民人均可支配收入和农牧民人均纯收入均比2010年翻一番。积极发展教育、卫生等社会事业，健全城乡统筹的就业服务体系，建成覆盖城乡居民的社会保障体系，完善以城乡低保为重点的社会救助体系，促进城乡社会服务一体化发展。加强和创新社会管理，用群众工作统揽信访维稳工作，推行“三一四全”、“三联六调”工作法，提高社会管理服务水平。深化平安创建，健全立体化社会治安防控体系，增强人民群众安全感。全面落实党的民族宗教政策，支持国防和军队建设，筑牢祖国北疆安全稳定屏障。

　　（四）实施文化引领工程，建设文明巴彦淖尔。文化承载历史，文明昭示未来。我们将加快推动文化大发展、大繁荣，建设独具魅力的民族文化大市。深入开展社会主义核心价值体系学习教育，将“总干”精神贯穿于经济社会发展各方面，凝聚加快发展的强大合力。加强文化基础设施建设，新建鸿雁大剧院和一批基层文化活动场所。实施重点文化惠民工程，完善四级公共文化服务网络。办好河套文化艺术节和研讨会，继续推进阴山岩刻申报世界文化遗产工作。扶持培育文化骨干企业，发展壮大传媒、影视、戏剧等文化产业。深度挖掘人文、历史文化资源，创作推出更多体现河套文化、乌拉特草原文化特色的精品力作。深入开展文明城市、文明村镇、文明行业等精神文明创建活动，全面提升公民道德素质，让现代文明之花在河套大地绚丽绽放。

　　（五）实施开放带动工程，建设活力巴彦淖尔。开放是发展的活力源泉，向北开放是我市最大的优势所在、潜力所在。加强甘其毛都口岸基础设施建设，大幅提高通关过货能力，年过货量力争突破5000万吨。规划建设20平方公里中蒙跨境经济合作区，培育新的境内外资源加工贸易基地。加强区域交流与合作，承接发达地区产业转移，全力支持非公有制经济发展，优化发展环境，打造诚信政府，吸引更多客商到我市投资兴业。大力推进科技创新，统筹抓好各类人才队伍建设，激发全社会创造活力和创业激情。按照中央和自治区部署，稳步推进各项改革，为赶超崛起提供体制机制保障。

　　各位代表，新的目标催人奋进，新的征程任重道远。我们坚信，在市委的坚强领导下，经过全市上下的共同努力，未来五年的宏伟蓝图一定会变成现实，巴彦淖尔的明天一定会更加美好！

　　三、2013年的工作

　　今年是全面贯彻党的十八大精神的第一年，也是新一届政府的开局之年，做好今年的工作意义重大。按照党的十八大、中央经济工作会议、自治区党委九届五次全委会议暨全区经济工作会议和市委三届五次全委（扩大）会议精神，本着实事求是、科学理性、积极进取的态度，确定今年全市经济社会发展的主要预期目标是：地区生产总值增长13%，力争达到15%；地方财政总收入增长11%以上，突破百亿元大关；固定资产投资增长14%，达到800亿元；社会消费品零售总额增长18%；城镇居民人均可支配收入增长15%；农牧民人均纯收入增长13%；城镇登记失业率控制在4%以内；完成节能减排目标任务。重点抓好八个方面的工作。

　　（一）抓迎会，促发展。今年全区第九次精神文明建设经验交流会在我市召开，借助这次全区的盛会，以会促建，推动巴彦淖尔经济、社会、文化、生态各个方面有新变化、大发展，展现巴彦淖尔的新形象、新面貌。以迎会办会为总抓手，集中精力办大事、抓重点、求突破。坚持市区共建，突出临河主城区，东西线并举，点线面结合，软硬件齐抓，高标准、高质量做好各项工作。进一步细化目标任务，责任到人，倒排工期，抢抓进度，所有项目要在6月底前完成。开展丰富多彩的精神文明创建活动，办好河套文化艺术节、河套文化主题展，办一届节俭务实、有亮点、有特色的文明盛会。

　　（二）抓工业，保增长。把抓投资、抓项目作为抓工业的重中之重，千方百计促进工业经济平稳增长。认真落实国家和自治区保增长的各项政策措施，从电价补贴、信贷融资等方面加大扶持力度，帮助停产半停产企业减负增效、渡过难关。加强工业经济监测调度，抓好煤电油运等生产要素保障。加大工业项目建设力度，全年实施重点工业项目180项，其中续建79项、新建101项，完成投资265亿元。包钢煤焦化、联邦制药四期及五期、太平矿业二期、燕京金川啤酒、圣泉生物质能源一期等项目建成投产。开工建设神华二期480万吨煤焦化、大中矿业100万吨脱氧球团、正大20万吨饲料、京东商城云计算、国电蒙电40兆瓦光伏电站、川井500千伏风电汇集站等项目，新增风电装机100万千瓦，光伏发电能力达到20万千瓦。推进河套酒业技改扩建、新湖和黄河工贸PVC、亚王热电联产及神华、盾安等5个自备电厂项目。抓好口岸和园区建设。启用甘其毛都口岸“五出五进”通道和“快捷通”系统，过货量突破1500万吨，争取过境铜精粉落地加工。组建巴彦淖尔国际商会，开展对蒙进出口贸易协调服务。完善园区各类专项规划，做好设施配套、绿化美化等工作，打造一批花园式企业和园区。完善巴彦淖尔经济技术开发区配套设施，提高承载能力。落实促进中小微企业发展的政策措施，建好临河、杭后中小企业创业园。启动巴彦淖尔经济开发区污水处理厂二期工程，前旗、中旗、后旗3个园区污水处理厂建成投用。牢固树立招商决定项目的理念，下大力抓好招商引资和项目引进，全年争取国内到位资金360亿元。

　　（三）抓“三农”，稳基础。加强农牧区基础设施建设。投资5亿元完成灌区排水改造工程3078公里，完成三年任务的34%。实施中低产田改造50万亩、土地整理45万亩、节水改造20万亩。依托天然林保护、京津风沙源治理等项目，大规模开展造林绿化，打造京藏高速、黄河堤防公路等宽林带精品工程，新增人工造林35万亩。落实好草原生态补奖等政策，严格实行草原禁牧、休牧、轮牧制度。完成乌兰布和沙漠综合治理17万亩。按照因地制宜、体现特色、方便群众的原则，规划建设67个新农村新牧区示范点，抓好县城周边和交通干道沿线村容整治，完成农村牧区危房改造3万户。新建农村牧区油路600公里，新增沼气入户3500户。提高农牧业生产效益。引导农民合理调整种植结构，扩大高效特色农作物种植比重。新增设施农业5万亩，发挥临河浩彤、杭后春光、五原隆兴昌等设施农业重点示范项目引领作用。强化技术培训、市场销售，提高设施农业利用率和收益率。新建、改扩建标准化规模养殖场300个，抓好富川现代肉羊园区、圣牧高科有机牧场等项目建设。提高农牧业服务保障水平，培育壮大专业合作组织，引导发展规模化经营，鼓励土地经营权向龙头企业、种养大户流转，新增土地流转50万亩。建立内蒙古西部农畜产品检测中心，完善农畜产品质量安全监管体系。强化动植物疫病防控，抓好气象、科技、信息、保险等服务。市和旗县区财政安排专项资金3.36亿元，增加4155万元，支持“三农三牧”，使农牧业的基础更稳，农牧区的面貌更新，农牧民的收入更高。

　　（四）抓三产，扩消费。加快旅游开发。编制黄河旅游发展规划，引进有实力的企业开发旅游资源，完善旅游景区配套设施建设。强化旅游宣传促销，发展农家乐、渔家乐、草原游等特色旅游，推进总干渠水上旅游项目建设。打造河套酒业、纳林湖、冬青湖、乌拉特部落等8个3A级以上旅游景区，新增4家三星级以上酒店。完善交通物流体系。交通完成投资35亿元，建成青山至乌根高勒、五原至刘召、海流图至五原、临河绕城线一级公路，开工建设临哈高速、临河至甘其毛都口岸一级公路，加快锡乌铁路、五原黄河大桥建设，完成临陕、陕东线整修改造。抓好B型保税物流园区配套设施建设，实现公路港通关运营。推进口岸物流综合监管区、巴运物流临河货运东站、秋林物流园区及各园区铁路专用线建设。改革和完善金融服务体系，积极支持金融企业做大做强，加快农村信用社股份制改造，稳步发展村镇银行和小额信贷机构。发挥各级投融资平台作用，扩大融资规模，解决发展资金短缺的问题。大力培植财源，加大税收征管力度，确保应收尽收。做好第三次全国经济普查工作，摸清经济底数。落实国家推广节能家电等政策，继续实施“万村千乡”市场工程，扩大城乡消费市场。

　　（五）抓城建，提品位。建好管好城镇是推进城镇化的基础。提高城镇品位，必须规划先行。按照套区突出农耕文化，牧区突出蒙元文化的原则，完善县城和集镇规划设计。完成朔方区总规和控制性详规修编。深度融入文化元素，做好城镇雕塑、游园、广场设计。大力推进双河区建设。金川大桥建成通车，启动先锋桥新桥和金川大桥南延道路、敕勒大街、先锋南路等主次干道项目，同步实施给排水、供电、绿化等配套工程。推进湿地保护和园林绿化。总干渠、永济渠、永清渠和镜湖、润昇湖、南湖、北海、迎宾湖、隆兴湖等湿地恢复工程全面完工。抓好街路、广场、公园等重要节点绿化，新增城镇绿地4200亩以上。完善城镇基础设施。实施棚户区改造69万平方米，其中临河35万平方米。完成临河新华街综合改造，打造横贯东西的25公里长街。打通河套大街。推进胜利路、金川大道等主要街路亮化美化。加大城镇出入口整治力度，重点抓好临陕、临磴等出口改造。解决好与群众生活息息相关的水、电、气、暖等问题，全市铺设管网340公里，新增集中供热面积200万平方米、天然气入户1.5万户，实现城镇生活垃圾处理全覆盖。抓好10个重点集镇建设，全面整治镇容镇貌。加强城市管理。进一步理顺市区两级城市管理体制，建设数字城管系统。开展市容环境集中整治，严厉打击私搭乱建行为。加强房地产和建筑业市场监管，整顿规范物业服务市场，提升物业服务水平。深化公交体制改革，优化公交布局，提高公交服务质量，方便群众出行。

　　（六）抓民生，办实事。民生连着民心，民心凝聚民力。越是困难的时候，越要高度关注民生。今年市和旗县区财政安排民生专项资金18.8亿元，增加4.3亿元。就业方面，新增城镇就业1.2万人，农村牧区劳动力转移就业16万人次，完成职业技能培训2.2万人，储备各类人才760名，发放小额担保贷款1.5亿元。社会保障方面，启动实施社会保障“一卡通”，城乡基本养老保险参保人数突破100万人；城镇职工、城镇居民医保年度最高支付限额分别由25万元提高到30万元、12万元提高到20万元；新农合人均筹资标准由300元提高到360元，政策范围内住院报销比例由75%提高到80%。社会救助方面，城市低保标准月人均提高50元，达到421元；农村牧区低保标准年人均提高360元，达到3085元；五保对象集中和分散供养标准年人均分别提高900元、500元，达到5818元、3315元；城镇“三无人员”、孤儿集中供养标准月人均分别达到1000元、1500元；支持残疾人事业发展，关心残疾人生活，开展白内障复明手术1000例，实施0～6岁贫困残疾儿童抢救性康复工程；实施集中连片、整村推进、移民扶贫“三大工程”，帮扶3万贫困人口脱贫。民生设施建设方面，新建保障性住房5499套，解决1.6万人的住房问题；实施安全饮水工程，让2万农牧民喝上放心水；建设自来水厂4个，提高城镇供水保障能力；新建2所市级老年人养护院和2所县级敬老院；加强殡葬管理，建成国家二级殡仪馆1个，提升殡葬服务水平。

　　（七）抓统筹，构和谐。统筹抓好各项社会事业，构建和谐巴彦淖尔。加大对社会事业的投入，市和旗县区财政安排教科文卫专项资金6.2亿元，增加1.6亿元。教育方面，落实学前教育三年行动计划，新建、改扩建公办幼儿园20所，学前三年适龄幼儿入园率达到80%以上。推进义务教育薄弱学校改造工程，完成市蒙古族中学和小学校舍改造。实施高中阶段免费教育，化解高中学校债务，筹建市职业技术学院，办好河套学院。稳定和加强师资队伍建设，着力提高教育教学质量。加大贫困学生资助力度，不让一个学生因贫辍学。科技方面，推进自治区级科技企业孵化器建设，支持企业开展技术创新，建成2家自治区级以上企业技术中心。实施科技惠民工程，加快科技成果推广普及，建设一批现代农牧业科技示范园区。文体方面，启动建设市体育馆，建成黄河水利文化博物馆、广电传媒中心、乌后旗国际那达慕体育场，新建基层文化活动室120个。实施广播电视“户户通”工程，让16万户农牧民免费收看卫星电视。举办沿黄公路自行车赛，建设城区室内健身活动场所2处、农村牧区体育健身中心10个。卫生方面，以BT方式投资10亿元，开工建设市医院新建项目。改建精神病院，完成7所旗县区卫生监督所、12所苏木乡镇卫生院和区域卫生信息平台建设。深化医药卫生体制改革，推进县级公立医院改革试点，扩大基本药物零差率销售范围。加强食品药品安全监管，确保群众饮食用药安全。抓好人口和计生工作，提高人口素质。创新社会管理，强化基层基础。完成19个苏木乡镇基础设施建设，每个旗县区新建1个2000平方米以上的高标准社区服务中心。建设标准化社区警务室27个，建成1个边防机动大队、4个边防派出所、1个边防检查站营房和2个爱民固边警务工作站。扎实开展社会矛盾排查化解，巩固信访维稳成效。落实安全生产责任制，坚决遏制重特大安全事故。做好民族和宗教工作，深化双拥共建。积极发展慈善事业、老龄事业，切实维护劳动者、妇女儿童合法权益。

　　（八）抓作风，树形象。空谈误国，实干兴邦。转作风就是要转到实干上来，转到谋发展、办实事、求实效上来。各级政府和机关工作人员要认真落实中央关于改进工作作风、密切联系群众的八项规定，思想上倡导心系群众、勇于创新、敢想敢干的作风；工作上倡导深入基层、雷厉风行、务实高效的作风；生活上倡导情趣高雅、勤俭节约、朴素正派的作风；纪律上倡导顾全大局、秉公用权、清正廉洁的作风，以良好的政风行风带动民风。大力倡导少开会、开短会、讲短话、发短文，用更多的时间研究问题、解决困难、指导工作。改革行政审批制度，提高行政效能，坚决整治不负责任、议而不决、拖而不办的机关顽疾，努力打造人民满意的服务型政府，以良好的形象赢得群众的信任和支持。

　　政府的权力是人民赋予的，必须为人民服务，对人民负责，受人民监督。要坚持依法行政，主动接受人大及其常委会的法律监督、政协的民主监督，认真办理人大代表建议和政协委员提案，广泛听取民主党派、工商联、无党派人士和老干部的意见。深入推进惩治和预防腐败体系建设，强化对重点领域和关键岗位人员的监管，努力做到干部清正、政府清廉、政治清明。

　　各位代表，奋斗成就伟业，实干铸就辉煌。让我们在市委的正确领导下，大力践行“总干”精神，锐意进取，扎实工作，为全面建成小康社会而奋斗，为共同创造全市人民更加美好幸福的未来而奋斗！
